# Cosmetogenomics unveiled: a systematic review of AI, genomics, and the future of personalized skincare

**DOI:** 10.3389/frai.2025.1660356

**Published:** 2025-11-10

**Authors:** Diala Haykal, Frédéric Flament, David Amar, Hugues Cartier, Arianne Shadi Kourosh, Dong Hun Lee, Christopher Rowland-Payne

**Affiliations:** 1 Centre Laser Palaiseau, Palaiseau, France; 2 L’Oréal Research and Innovation, Clichy, France; 3 Centre Médical Saint Jean, Arras, France; 4 Department of Dermatology, Harvard Medical School, Boston, MA, United States; 5 Department of Environmental Health, Harvard TH Chan School of Public Health, Boston, MA, United States; 6 Seoul National University Hospital, Seoul National University College of Medicine, Seoul, Republic of Korea; 7 The London Clinic, London, United Kingdom

**Keywords:** genomics, proteomics, predictive analysis, precision medicine, artificial intelligence, skin aging

## Abstract

**Introduction:**

The integration of genomics, proteomics, and artificial intelligence (AI) is shaping the approach to personalized skincare and aesthetic dermatology, moving from generalized protocols toward precision-based interventions.

**Objective:**

To systematically review the emerging field of cosmetogenomics, focusing on how AI and multi-omics technologies are enabling personalized dermatologic treatments, and to critically evaluate the strength, scope, and limitations of current evidence.

**Methods:**

We conducted a systematic review in accordance with PRISMA 2020 guidelines. PubMed, Scopus, and Embase databases were searched for articles from January 2012 to April 2025 using Boolean combinations of terms including [“cosmetogenomics” OR “AI in dermatology” OR “personalized skincare” OR “multi-omics dermatology”] AND [“SNP” OR “genomics” OR “proteomics”]. Eligible studies included peer-reviewed clinical or ex vivo research involving human subjects and reporting measurable dermatologic outcomes related to genomics, single nucleotide polymorphisms (SNPs), AI tools, or proteomics. Study quality was assessed using the JAMA Users’ Guides to the Medical Literature quality scheme.

**Results:**

From 403 screened articles, 74 met inclusion criteria. Of these, 22 were randomized controlled trials (RCTs, Level I evidence), 35 observational studies (Level II), and 17 conceptual or expert opinion papers (Level III). AI and genomics were found to enhance skincare personalization by identifying SNPs associated with collagen degradation, oxidative stress, and inflammation. AI-powered platforms integrate these insights with imaging, lifestyle data, and digital twins to optimize interventions ranging from topical regimens to laser and injectable treatments. However, a significant proportion of studies were exploratory, with limited geographic diversity and underrepresentation of darker skin phototypes. No quantitative synthesis (meta-analysis) was performed due to heterogeneity in outcome measures, though hydration, elasticity, and pigmentation outcomes may permit such analysis in future work.

**Conclusion:**

AI-driven cosmetogenomics is advancing dermatology into a predictive, personalized era. While the evidence base is expanding, clinical translation requires stronger validation, ethical safeguards, and regulatory oversight. This field holds significant promise for enhancing treatment efficacy, patient satisfaction, and long-term skin health. Broader validation, greater diversity in study populations, more transparent methodologies, and expanded ethical safeguards, including genetic discrimination risks, data ownership, and cross-border data transfer, are necessary before widespread clinical integration.

## Introduction

The convergence of genetic research, artificial intelligence (AI), and dermatology is reshaping the landscape of aesthetic medicine. Traditional skincare regimens have relied on generalized formulations, but advances in genetic analysis now enable treatments aligned with an individual’s biological profile (Haykal, 2024a). DNA-based dermatological assessments, combined with AI-driven insights, represent a potentially more precise approach that considers genetic predispositions to collagen degradation, oxidative stress, inflammatory responses, and barrier function (Haykal, 2025a; Papaccio et al., 2022; Hussein et al., 2025; Chmielewski and Lesiak, 2024). Personalized skincare informed by genomics would help reduce trial-and-error in clinical care, while AI technologies further integrate genetic, environmental, and lifestyle factors to generate dynamic, evidence-based recommendations (Su et al., 2024; Ho et al., 2020; Pandey and Gupta, 2024; Molla and Bitew, 2024; Giansanti, 2024).

This precision-oriented perspective has given rise to the emerging concept of cosmetogenomics. Derived from “cosmetics” and “genomics,” cosmetogenomics refers to the study of how an individual’s genetic makeup influences their response to cosmetic and aesthetic dermatological interventions. While much literature currently overlaps with broader dermatogenomics, which traditionally emphasizes disease diagnostics and therapeutic strategies, cosmetogenomics is distinguished by its focus on aesthetic outcomes such as aging, pigmentation, hydration, and elasticity. By centering on enhancement and maintenance of skin health and appearance, rather than treatment of disease, cosmetogenomics represents a complementary but distinct dimension within precision dermatology.

Despite these promising directions, integrating genetic analysis into dermatology presents significant challenges. Key issues include data privacy, ethical considerations, and the clinical validation of AI-driven genomic predictions (Sengupta, 2023; Mondal and Mondal, 2024). Moreover, the interplay between genetics, epigenetics, and environmental factors complicates the translation of genomic findings into predictable skincare outcomes (Andersen and Millar, 2021; Landau et al., 2024). As research progresses, striking a balance between innovation and responsible implementation will be critical.

We performed a systematic review to evaluate the current landscape of cosmetogenomics, emphasizing the methodological rigor, evidence quality, and ethical considerations necessary for responsible clinical adoption.

## Methods

This systematic review was conducted in accordance with the PRISMA 2020 guidelines. The objective was to synthesize available evidence regarding the application of genomics and AI in personalized skincare, a field referred to as cosmetogenomics, with an emphasis on distinguishing its aesthetic focus from broader dermatogenomics.

We carried out a comprehensive search of PubMed, Scopus, and Embase databases for articles published between January 1, 2012, and April 1, 2025. Boolean combinations of search terms were applied, including [“cosmetogenomics” OR “AI in dermatology” OR “personalized skincare” OR “multi-omics dermatology”] AND [“SNP” OR “genomics” OR “proteomics”]. Medical Subject Headings (MeSH) were used where available. Only articles published in English were considered. Eligible studies were peer-reviewed original articles or systematic reviews involving human subjects or ex vivo skin models, addressing AI, genomics, SNPs, proteomics, or multi-omics in dermatology and reporting measurable dermatologic or cosmetic outcomes. Studies were excluded if they focused exclusively on animal models, addressed non-dermatological conditions, or lacked specific skin-related outcomes.

Two reviewers independently screened all titles and abstracts. Full texts of potentially relevant articles were retrieved and assessed for eligibility. Disagreements were resolved by discussion or by consulting a third reviewer. Data extraction was performed independently using a standardized template, capturing study type, population characteristics, technologies employed (genomic, proteomic, AI), intervention type, outcome measures, and key findings. Methodological quality was assessed using the JAMA Users’ Guides to the Medical Literature rating scheme, ranging from Level I (randomized controlled trials) to Level V (expert opinion or conceptual articles).

**Figure 1:**
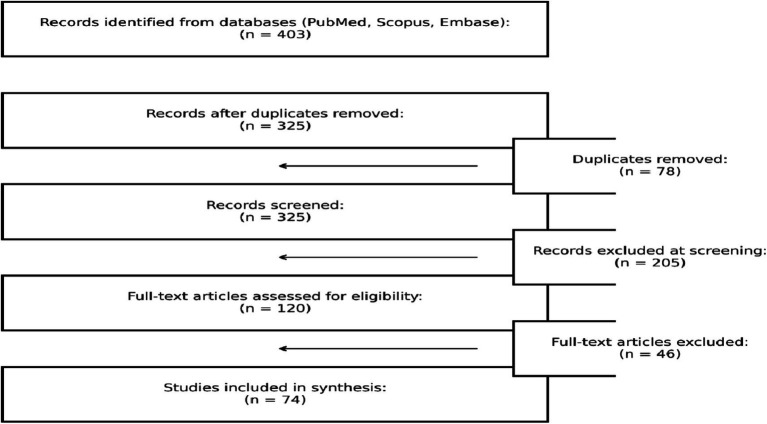
Flowchart.

**Figure 2:**
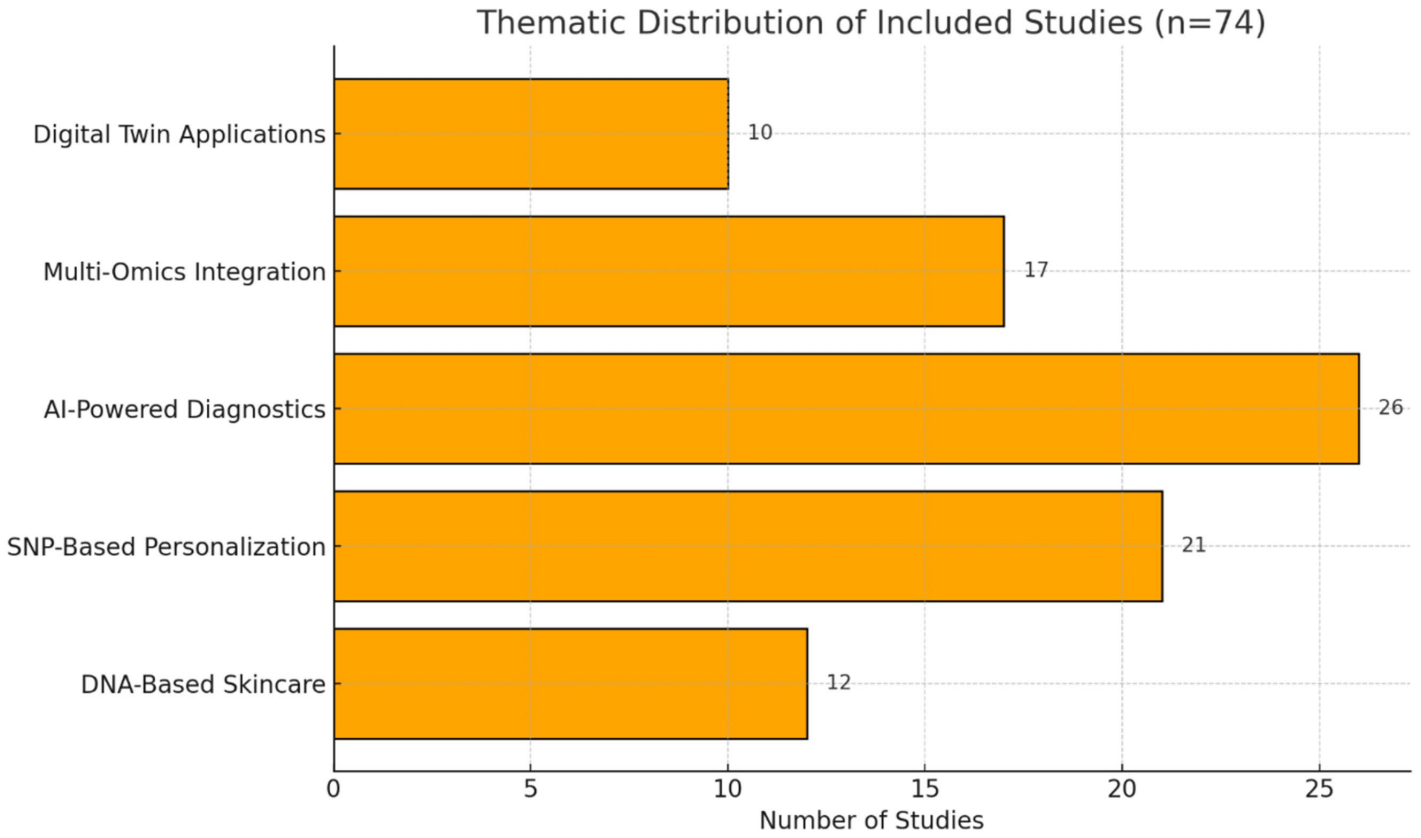
Thematic distribution of included studies.

A total of 403 articles were initially identified. After duplicate removal and screening of titles and abstracts, 325 studies remained for further consideration. Among these, 120 full-text articles were assessed in detail, and 74 met eligibility criteria for inclusion. The article selection process is outlined in Figure 1. Due to heterogeneity in study designs and reported outcomes, a qualitative thematic synthesis was performed. Studies were grouped based on their primary focus (e.g., SNPs, AI tools, multi-omics, digital twins); however, several studies addressed multiple thematic areas and were therefore represented in more than one category. (Figure 2).

## Results

The studies included in this review provide a multifaceted understanding of how AI and genomics are being leveraged in dermatology to individualize care and improve patient outcomes. The findings span a range of innovations from genetic risk stratification to advanced modeling and integrative diagnostics. While the findings highlight emerging opportunities, they also reveal important limitations in methodological rigor, population diversity, and long-term validation. The following subsections highlight the major themes that emerged from the evidence synthesis.

### The potential of DNA-based skincare

Advances in genomic sequencing have facilitated the identification of genetic variations that influence skin aging, barrier function, and response to environmental stressors (Hussein et al., 2025; Russell-Goldman and Murphy, 2020; López-Otín et al., 2023). Genetic insights provide an opportunity to personalize skincare interventions, moving beyond traditional one-size-fits-all approaches (Markiewicz and Idowu, 2022). For instance, individuals with genetic markers indicating reduced antioxidant defenses could benefit from antioxidant-enriched formulations, while those with inflammatory predispositions may respond to anti-inflammatory compounds. Similarly, barrier dysfunction markers may indicate a need for barrier-restoring ingredients. Although promising, these applications remain largely hypothesis-driven, with limited large-scale clinical validation. Moreover, evidence has been disproportionately drawn from lighter phototypes and developed market populations, which limits generalizability.

The application of DNA-based insights extends beyond topical formulations. Understanding genetic variations in collagen synthesis and degradation can inform decisions regarding procedural dermatology, optimizing treatment choices for injectables, laser resurfacing, and regenerative therapies (Dutra Alves et al., 2025). In the future, AI-driven genomic analysis could enable real-time, adaptive treatment plans, dynamically adjusting recommendations based on continuous skin assessments (Alowais et al., 2023).

### The role of single nucleotide polymorphisms in personalized skincare

Single nucleotide polymorphisms (SNPs) are among the most studied genetic variations in dermatology research (Haykal, 2025a). SNPs in genes such as MMP1 (collagen degradation) and SOD2 (oxidative stress management) have been linked to differential skin aging processes (Sepetiene et al., 2023). These findings provide a rationale for individualized strategies, such as collagen-stimulating treatments or antioxidant-focused regimens.

However, SNP-based personalization remains an emerging field. Outcomes are influenced by environmental exposures, lifestyle factors, and epigenetic modifications, which complicates direct clinical translation. Standardized protocols and multi-center validation studies are needed to determine the robustness of SNP-informed interventions (Markiewicz and Idowu, 2018; Haykal, 2025b).

### AI-powered tools in personalized skincare

Artificial intelligence (AI) is transforming personalized skincare by enabling real-time analysis and dynamic treatment recommendations. AI-powered platforms integrate clinical data, environmental influences, and imaging-based skin assessments to provide highly individualized skincare protocols (Ho et al., 2020; Rezayi et al., 2022; Haykal, 2024b). Machine learning algorithms analyze skin texture, hydration levels, pigmentation patterns, and wrinkle formation to detect subtle changes over time, allowing for early intervention and adaptive skincare strategies (Quazi, 2022). AI is also transforming diagnostic dermatology by enhancing image-based skin assessments (Kania et al., 2024). AI-driven imaging tools assess hyperpigmentation, fine lines, and overall skin structure, offering quantifiable insights that refine skincare product selection and procedural recommendations (Li et al., 2022). Additionally, AI-powered chatbots and virtual consultations provide real-time, data-driven skincare guidance, making personalized skincare more accessible beyond traditional clinical settings (Chakraborty et al., 2024; Jing et al., 2024). Beyond skincare recommendations, AI is enhancing treatment monitoring, tracking real-time changes in skin health due to seasonal variations, hormonal shifts, and environmental factors (Haykal, 2025c). A key advancement of this transformation is the integration of omics-based diagnostics with advanced imaging analysis, both within clinical settings and through digital touchpoints such as smartphones and teleconsultations. This continuous assessment allows for real-time adaptation of skincare recommendations, ensuring treatments evolve based on a patient’s biological profile and environmental exposures. Grounded in cosmetogenomics, this approach enhances precision dermatology by combining genetic, proteomic, and epigenetic insights with AI-based image analysis, optimizing both at-home skincare and in-clinic interventions (Tong et al., 2024).

Recent advancements in AI-driven personalized skincare devices contributed to a shift toward tailored treatments (Flament et al., 2023). These innovations illustrate how AI-driven formulation technologies are bridging the gap between genetic insights and real-world applications, delivering customized skincare products at the consumer level. Future developments may integrate multi-omics data including genomics, proteomics, and microbiome analysis for even more precise, dynamically adaptive skincare solutions. To enhance adherence, predictive perception engines, AI systems that analyze user behavior, preferences, and feedback to anticipate future choices, personalize recommendations by ensuring products not only address skin concerns but also align with user preferences in texture and scent, creating a seamless and enjoyable skincare routine (Rodan et al., 2016). AI further extends its capabilities to detect unexpressed skin concerns, recognizing subtle indicators that may affect quality of life, even if users do not explicitly report them (Flament et al., 2024). By utilizing Generative Adversarial Networks and Virtual Try-On technologies, AI adapts treatment intensity and frequency to meet individual needs dynamically (Despois et al., 2020).

While these tools are expanding accessibility and personalization, many remain at early proof-of-concept stages and lack independent validation. Furthermore, training datasets often underrepresent diverse skin phototypes, raising concerns about generalizability.

### AI-based tools in genomics and proteomics

AI integration with genomic and proteomic datasets is being explored for predictive modeling of skin health trajectories. Proteomic analyses have identified biomarkers related to hydration, elasticity, and pigmentation, while machine learning models aim to combine these with genomic profiles to forecast outcomes and inform interventions. To illustrate, AI-driven genomic analysis can rapidly identify genetic markers associated with collagen degradation, oxidative stress response, and skin barrier integrity, allowing dermatologists to design targeted skincare regimens (Gupta and Margolis, 2020; Van Doren, 2015; Gupta et al., 2023). Beyond genomics, AI is transforming proteomics and biomarker discovery, analyzing molecular-level skin responses to determine early indicators of skin aging, inflammation, and hydration loss (Guo et al., 2022). Deep learning models process vast datasets to correlate genetic predispositions with clinical skin characteristics, refining treatment precision in aesthetic procedures such as laser resurfacing, bio-stimulatory injectables, and regenerative therapies (Jeong et al., 2022).

AI’s ability to integrate multi-omics data (genomics, proteomics, and metabolomics) enhances dermatological research by identifying complex skin-aging pathways and predicting long-term skincare outcomes (Pun et al., 2023; Nakajima et al., 2024; Dessì et al., 2024). This AI-powered approach ensures that genetic insights are viewed in the context of an individual’s full biological profile, bringing precision dermatology closer to real-world clinical application (Bohr and Memarzadeh, 2020).

The integration of omics-based diagnostics with AI-driven imaging and real-time teleconsultations bridges digital health with dermatology, allowing skincare regimens to evolve dynamically. This approach signifies a transition from static recommendations to real-time, data-driven skincare solutions, marking the intersection of AI, dermatology, and cosmetogenomics.

### Digital twins, genomics, and predictive dermatology

The integration of AI-driven digital twins with genomic insights is being explored as a potential tool in predictive dermatology, with the aim of supporting more refined approaches to personalized skincare (Haykal, 2025b). A digital twin is a virtual representation of an individual’s skin, using genetic data, multi-omics profiling (genomics, proteomics, metabolomics, and microbiome analysis), environmental exposures, and lifestyle factors (De Domenico et al., 2025). Unlike traditional static genetic assessments, digital twins dynamically evolve, continuously updating based on new inputs such as UV exposure, pollution, hydration levels, hormonal fluctuations, and treatment responses (Papachristou et al., 2024; Meijer et al., 2023). By leveraging genomics, these models can predict an individual’s predisposition to collagen degradation, oxidative stress susceptibility, inflammatory responses, and barrier dysfunction, enabling highly targeted skincare and aesthetic interventions (Li et al., 2025; Wang et al., 2024). AI-powered machine learning algorithms correlate genetic markers with real-world skin responses to forecast aging patterns, treatment efficacy, and risk factors for adverse reactions (Yang and Kar, 2023; Zhavoronkov et al., 2019). In procedural dermatology, digital twins could optimize laser resurfacing parameters based on genetic predisposition to pigmentation disorders or determine the ideal bio-stimulatory injectable protocol based on collagen synthesis capacity (Haykal, 2025b). This approach marks a potential shift from reactive to proactive and preventive dermatology, allowing both clinicians and patients to anticipate skin concerns before they manifest, ultimately enhancing treatment precision, efficacy, and patient satisfaction.

## Discussion

The findings of this review suggest that AI and genomics are contributing to new approaches in dermatology, particularly in personalization and prediction of outcomes. Reported innovations range from genetic markers that may guide topical formulations to early-stage explorations of digital twins. However, enthusiasm should be tempered by recognition of the modest evidentiary base, the predominance of exploratory studies, limited population diversity, and underdeveloped ethical frameworks. Addressing these gaps will be essential for responsible clinical translation.

### Clinical applications of AI and genomics in aesthetic dermatology

AI is transforming aesthetic dermatology by enhancing precision, safety, and predictive modeling in non-invasive treatments. While AI-powered tools have already optimized personalized skincare formulations, their impact extends beyond product recommendations to procedural interventions such as laser therapy, injectables, and regenerative treatments (Haykal, 2024a). AI-driven algorithms analyze a patient’s genetic profile, skin histology, and real-time imaging data to refine treatment settings, product choices, and post-procedure care, ensuring predictable and highly personalized outcomes (Schork, 2019).

In laser dermatology, AI is being integrated into energy-based devices to automate treatment parameter selection, reducing the risk of post-inflammatory hyperpigmentation or excessive collagen remodeling in patients with genetically predisposed sensitivity (Haykal, 2025d). AI-powered software also assesses vascularity, melanin content, and skin hydration levels in real-time, adjusting laser fluence and pulse duration for optimized resurfacing outcomes. For example, a 28-year-old woman with a TNF-*α* polymorphism predisposing to heightened inflammatory responses underwent a personalized, low-fluence non-ablative laser protocol, supported by an antioxidant-rich skincare regimen. Regular imaging follow-up assessments demonstrated decreased erythema and enhanced skin texture (Haykal, 2025a).

For injectable treatments, AI is advancing predictive analytics to improve hyaluronic acid filler selection, toxin placement, and collagen-stimulating treatments. Deep learning models can predict how facial structures will change post-treatment based on genetic predispositions to collagen degradation (MMP1 mutations) or inflammatory responses (Freitas-Rodríguez et al., 2017). This assisted clinicians in selecting the most appropriate filler, injection depth, and volume to achieve natural and long-lasting results. In another instance, a 50-year-old man with an MMP1 gene variant indicating accelerated collagen degradation received biostimulatory injectables rather than hyaluronic acid fillers. Evaluations at 1, 3, and 6 months showed progressive structural improvement and skin elasticity. Moreover, AI is mitigating adverse events by detecting early markers of inflammation, vascular compromise, or delayed hypersensitivity reactions in injectable treatments. By integrating real-time imaging and patient history, AI-powered risk assessment tools provide preemptive alerts for potential complications, improving treatment safety and patient satisfaction. A third case involved a 45-year-old woman with SOD2 polymorphisms suggesting poor oxidative stress management. A preparatory antioxidant regimen was introduced six weeks before her fractional CO₂ laser procedure. Post-treatment follow-up revealed enhanced healing and reduced erythema, highlighting the predictive value of genomic profiling in procedural planning (Mansoor et al., 2025; Velarde et al., 2012).

**Figure 3:**
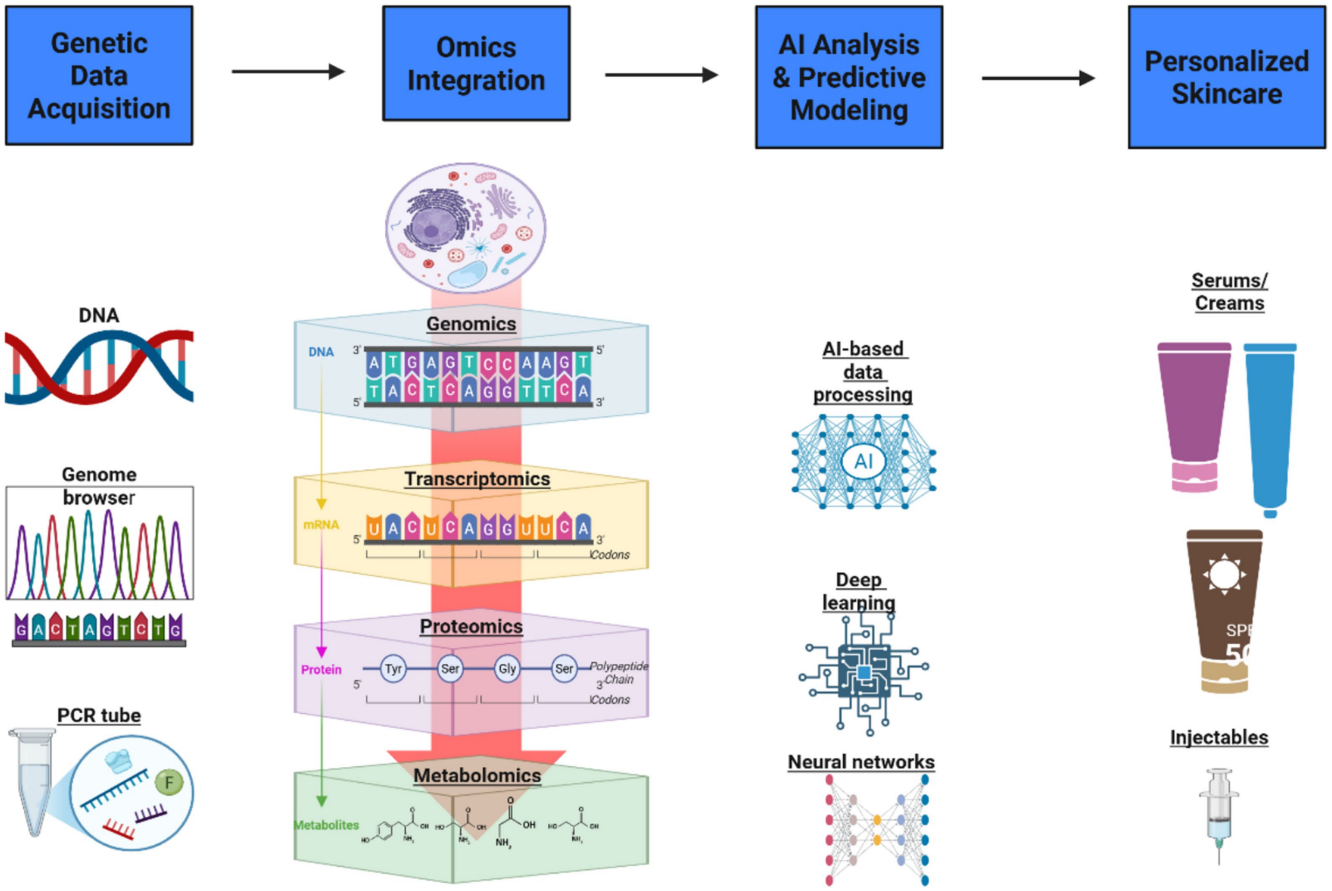
Integrative pipeline for AI-driven personalized skincare.

These advancements mark a paradigm shift in aesthetic dermatology, where AI-driven predictive modeling enables highly personalized, risk-optimized treatments that align with individual genetic and phenotypic characteristics. By leveraging data-driven insights, automated procedural adjustments, and real-time risk analysis, AI would help standardize aspects for precision, efficacy, and patient-centered care in modern aesthetic medicine. Figure 3 illustrates the integrative pipeline from genetic data acquisition through AI-powered analysis to the development of personalized skincare protocols.

### Level of evidence overview

To contextualize these clinical insights, we evaluated the strength and quality of the supporting evidence. Among the 74 studies reviewed, 22 were randomized controlled trials (Level I), demonstrating high internal validity and often investigating the clinical efficacy of SNP-guided interventions and AI-based customization in skincare. These RCTs reported statistically significant improvements in skin hydration, elasticity, and texture when treatment was personalized. Approximately 35 studies were observational (Level II), including prospective and retrospective cohorts exploring AI-assisted diagnostics, omics integration, and personalized regimens. While they provided strong real-world evidence, they frequently lacked randomization and blinding.

The remaining 17 studies were categorized as Level III evidence, comprising expert consensus statements, pilot modeling projects, and conceptual papers introducing digital twin strategies and predictive simulation tools. Though conceptually robust, these studies had limited empirical validation.

Overall, 57% of the studies (Levels I and II) offered moderate to high quality evidence, while 23% were early-phase or exploratory. Many of the higher-quality studies demonstrated consistent outcome improvements aligned with genetic profiles or AI analysis. In addition, limited geographic diversity and underrepresentation of darker phototypes restrict generalizability. Yet, limitations included lack of population diversity, inconsistent outcome measures, and limited follow-up.

### Ethical and data security concerns

The ethical challenges of integrating AI and genomics into dermatology extend beyond data privacy, algorithmic transparency, and bias (Clayton et al., 2023). Risks include potential genetic discrimination in employment or insurance contexts if genomic information is misused. Issues of data ownership and consent are particularly salient, as for commercial purposes collection and control by different players of skin genomic data increase drastically, raising concerns about secondary use without explicit patient approval (Kaye, 2012; Sanderson et al., 2017).

Additionally, cross-border data transfer creates regulatory and jurisdictional challenges, as protections vary widely (e.g., GDPR in Europe vs. HIPAA in the United States), potentially exposing individuals to vulnerabilities (Metta et al., 2024; Kovari, 2024; Chen et al., 2022). Ensuring algorithmic transparency and explainability remains critical to prevent opaque decision-making and to build patient and clinician trust. Equity considerations are also central, since underrepresentation of diverse phototypes in genomic and AI datasets may exacerbate disparities in outcomes (Chassang, 2017; Mennella et al., 2024; Faheem et al., 2025). Addressing these challenges through robust ethical frameworks, regulatory oversight, and inclusive study design is essential for responsible clinical adoption of cosmetogenomics.

### The future of DNA-based aesthetic dermatology

Looking ahead, advancements in DNA-based skincare and aesthetic dermatology lies in multi-omics integration, combining genomics with proteomics, metabolomics, and microbiome analysis for a more comprehensive understanding of skin health (Dessì et al., 2024; Li et al., 2023). AI and machine learning could further enhance this field by refining predictive models and generating truly personalized skincare algorithms (Elder et al., 2021; Mataraso et al., 2025). In parallel with these diagnostic and predictive advances, therapeutic innovation is rapidly evolving. Emerging technologies such as gene editing and targeted epigenetic modulation offer the potential to directly influence skin biology, transitioning from surface-level product application to interventions that operate at the molecular level (Baker and Hayden, 2020; Dermitzakis et al., 2025).

While the integration of AI and multi-omics approaches offers promising directions in aesthetic dermatology, it is important to acknowledge current limitations. These include the high cost and limited accessibility of SNP and omics testing, the lack of standardized protocols for AI-driven dermatogenomic tools, and challenges in clinical validation and reproducibility. These technical and systemic barriers must be addressed to ensure equitable and reliable implementation in real-world dermatology practice (Table 1).

**Table 1:** Summary of some studies.

Reference/Study	Population	Intervention/Study design	Primary outcomes	Quality of evidence (1–5)
Sepetiene et al. (2023)	Subjects with genetic polymorphisms	Subjects with genetic polymorphisms	Subjects with genetic polymorphisms	2
Flament et al. (2023)	Women of various ages and skin types (cross-sectional)	Cross-sectional imaging analysis of selfie-based AI scoring (JEADV)	Improved facial sign analysis across diverse skin types	2
Dessì et al. (2024)	Participants in integrative multi-omics skin study	Multi-omics integration in skincare personalization (Metabolites)	Identified aging/inflammatory biomarkers from omics integration	3
Gupta and Margolis (2020)	Patients with FLG mutations and barrier dysfunction	Review of filaggrin gene mutations and clinical implications	Identified FLG mutations affecting skin barrier, supporting personalized interventions	3
Haykal (2025a)	Conceptual framework on digital twins in skincare	Perspective on digital twin models in dermatology	Theoretical model for AI-driven digital twin simulation in dermatology	5

Beyond individual skincare, AI and genomics will reshape procedural dermatology by optimizing non-invasive treatments such as laser therapy, injectables, and regenerative medicine (Haykal et al., 2024). Real-time AI-based genomic feedback could adjust laser parameters according to an individual’s collagen synthesis capacity or determine the most suitable dermal filler based on genetic predisposition to inflammation (Haykal, 2025d). These innovations bridge the gap between precision medicine and aesthetic dermatology, ensuring safer and more effective treatments tailored to each patient’s unique biology (Johnson et al., 2021; Marques et al., 2024).

The intersection of genomics and AI marks a pivotal evolution in aesthetic dermatology. While early results are promising, widespread clinical integration depends on rigorous validation, equitable access, and ethical safeguards. Continued research and collaboration between dermatologists, data scientists, and regulatory bodies will be essential to fully realize the potential of personalized, precision-based skincare and interventions.

## Conclusion

The integration of DNA analysis into aesthetic dermatology represents a promising but still evolving frontier. The ability to personalize skincare based on genetic predispositions offers potential benefits, yet significant scientific, ethical, and practical challenges remain before widespread adoption is feasible. AI-driven tools may refine personalized approaches by supporting deeper analysis and adaptive treatment recommendations, while SNP analysis, proteomics, and predictive modeling can contribute to more precise interventions. However, the current evidence base is modest, geographically limited, and often exploratory.

Future research should prioritize the development of standardized dermatogenomic databases, independent validation of AI-driven protocols, and greater inclusion of diverse populations. Equally important are robust ethical safeguards addressing genetic discrimination, data ownership, and cross-border governance. With these measures in place, genetic-based skincare could move from theoretical innovation toward clinically validated, evidence-based practice. AI-driven genomic analysis, machine learning, and predictive models have the potential to enhance dermatology, but progress must be grounded in transparency, reproducibility, and equity. The intersection of AI, genomics, and molecular science offers opportunities for advancement, provided implementation is cautious, ethical, and scientifically rigorous.
