# Supplementary material for: Cosmetogenomics unveiled: a systematic review of AI, genomics, and the future of personalized skincare
Source: Front Artif Intell. 2025 Nov 10;8:1660356. doi: 10.3389/frai.2025.1660356 (PMC12640980; doi:10.3389/frai.2025.1660356)
Supplement: Supplementary file 1 [file Data_Sheet_1.docx]

**Continuing Professional Development (CPD)**

**Learning Objective**

After completing this CPD activity, readers should be able to:
Understand how artificial intelligence (AI) and genomics intersect in the field of cosmetogenomics to enable personalized dermatological care, recognize key genetic markers relevant to skin aging and inflammation, evaluate the clinical use of digital twin models, and reflect on the ethical considerations surrounding data use in AI-driven skincare.

**CPD Questions**

**1. What is the main purpose of integrating artificial intelligence (AI) into cosmetogenomics?**
A. To create universal skincare routines for clinical trials
B. To produce real-time virtual skin avatars for marketing
C. To automate manufacturing of cosmetic products
D. To personalize dermatological treatments using genomic and phenotypic data
E. To eliminate the need for dermatological consultations

**2. Which gene-related single nucleotide polymorphism (SNP) is most closely associated with impaired oxidative stress defense in the skin?**
A. MMP1
B. FLG
C. SOD2
D. TNF-α
E. IL-6

**3. In the context of dermatology, what is a digital twin?**
A. A holographic model used in aesthetic product display
B. A second biopsy sample taken for comparison
C. A virtual model of a patient's skin integrating genetic, environmental, and clinical data
D. A mirrored skin image used for pre-treatment simulations
E. A 3D rendering used only in surgical planning

**4. What is a key ethical concern when applying AI and genomics in dermatology?**
A. The reduced effectiveness of standard treatments
B. The inability to patent personalized skincare formulations
C. Misuse of personal genetic data and lack of regulatory safeguards
D. The inability of AI tools to analyse skin colour
E. Oversaturation of the skincare market

**5. How can AI-based SNP analysis be used to inform procedural dermatology?**
A. By guiding post-procedure antibiotic prescriptions
B. By predicting patient responsiveness to sunscreen
C. By identifying genetic sensitivities to inflammation and adjusting laser settings accordingly
D. By assessing emotional readiness for cosmetic treatments
E. By eliminating the need for clinical photography

**Answers:**
1–D, 2–C, 3–C, 4–C, 5–C
